# Prevention and Treatment of Chemotherapy-Induced Alopecia

**DOI:** 10.5826/dpc.1003a74

**Published:** 2020-06-29

**Authors:** Alfredo Rossi, Gemma Caro, Maria Caterina Fortuna, Flavia Pigliacelli, Andrea D’Arino, Marta Carlesimo

**Affiliations:** 1 Department of Hematology, Oncology and Dermatology, Sapienza University of Rome, Italy

**Keywords:** hair loss, chemotherapy, treatment, alopecia

## Abstract

**Background:**

Chemotherapy-induced alopecia (CIA) is one of the most dramatic side effects of chemotherapy. Currently no guidelines are available for its prevention and treatment. Several devices and drugs are used, but results are often disappointing.

**Aims:**

Our aim is to analyze drugs and devices proposed in the literature for prevention and treatment of CIA induced by cytotoxic drugs and to discuss the evidenced-based opinion.

**Methods and Results:**

Scalp cooling is the only agent that has been approved by the US Food and Drug Administration for CIA prevention. Minoxidil and bimatoprost should not be used during chemotherapy administration, but they can be used after chemotherapy discontinuation to obtain greater regrowth.

**Conclusions:**

Therapy should always be modulated for the patient and no fixed protocol should be used. Trichoscopy and trichogram could be useful tools in supporting this treatment.

## Introduction

Chemotherapy-induced alopecia (CIA), one of the most dramatic side effects of chemotherapy [1], occurs in about 65% of patients receiving cytotoxic drugs [2]. The hair-shaft shedding can occur from a few days to weeks after the beginning of chemotherapy, with different shedding patterns depending on the severity of the insult. Specifically, telogen effluvium is associated with mild to moderate damage, while dystrophic anagen effluvium follows severe damage.

No guidelines are available for the prevention and treatment of CIA. Several devices and drugs are used, but the results are often disappointing and the scientific rationale for their use is unclear [3].

To undertake successful management, it is important to distinguish among CIA induced by cytotoxic drugs, CIA induced by targeted therapy, and CIA induced by hormonal therapy. Each of these types is different in clinical aspect and pathogenetic mechanism, so treatment should be different too.

In this review we focus only on CIA induced by cytotoxic drugs. These agents are still largely used, and they are associated with the most extreme type of alopecia, which occurs in a high percentage of the cases. Moreover, all these drugs, although with different mechanisms, work by inducing apoptosis of rapidly proliferating cells [2,4]. This allows us to use therapeutic strategies that can be common to all of them. This approach is not possible with CIA induced by targeted therapies or hormonal treatments. Separate works should address those treatments.

As already stated, cytotoxic drugs all have as their main targets rapidly proliferating cells. Keratinocytes of the hair follicle matrix are highly proliferative during the anagen phase, and together with the pigmentary system they are very sensitive to toxins and drugs [2,4]. Instead, catagen and telogen phases are mitotically less active, so they are protected from chemotherapy toxicity [2,4]. Normally up to 90% of scalp hairs are in the anagen phase; for this reason hair loss is usually massive. Even if this kind of alopecia is generally reversible in 3 to 6 months, and permanent alopecia is rare, this side effect has a considerable psychological effect [2,4,5].

Our aim is to analyze drugs and devices proposed in the literature for the prevention and treatment of CIA induced by cytotoxic drugs and to discuss the evidenced-based opinion.

It is important to distinguish between drugs and devices aimed to prevent alopecia (such as scalp cooling) and drugs and devices that sustain hair regrowth.

## Scalp Cooling and Topical Vasoconstrictors

Scalp-cooling devices refrigerate the scalp with consequent local vasoconstriction and reduction of drug inflow to the hair follicles. It is largely used, and alopecia prevention rates are reported to be between 50% and 80% [6–8]. Recently, this device was approved by the US Food and Drug Administration as the only efficient agent preventing CIA [9]. Patient compliance is high, even though headache, discomfort, nausea, and xerosis may occur [10]. This device is recommended in patients affected by solid tumors undergoing chemotherapeutic protocols associated with a high risk of developing CIA. Meanwhile, patients receiving platinum derivatives have severe peripheral neuropathies which limit their tolerance to cold [11]; for this reason scalp cooling should be avoided in these patients. Moreover, the risk of scalp metastasis prohibits the use of scalp cooling in cases of hematological tumors [12]. Finally, this device is contraindicated in cold agglutinin disease, cryoglobulinemia, and posttraumatic cold injury because of the risk of triggering local or generalized attack [13,14].

Soref and Fahl evaluated the efficacy of topical vasoconstrictors epinephrine and norepinephrine in preventing chemotherapy- and radiotherapy-induced alopecia in rats [15]. Induction of hypoxia signal by local vasoconstriction preserves hair follicle cells and reduces the amount of drug reaching hair follicle [16]. The use of vasoconstrictors 3 times a day leads to effective and long-lasting vasoconstriction.

In humans, when applied applied on nasal mucosae for a long time, topical vasoconstrictors could induce rhinitis medicamentosa that may present with inflamed, dry mucosa prone to bleeding, edema, and associated insomnia [17,18]. It is reasonable to hypothesize that no risk of thinning and bleeding exists in cases of scalp application, since scalp is thicker than mucosa and has a different vascularization.

The limitation of scalp cooling is that its action is confined to the application time, which usually coincides with the drug infusion. We know that the half-life of chemotherapeutic drugs is longer than their infusion time; for this reason toxic effects are prolonged too. Scalp refrigerating and consequent vasoconstriction limited to infusion time could not prevent toxic effects occurring during the weeks from one infusion to another. Meanwhile, daily topical application of vasoconstrictors could overcome this problem to ensure better results in preventing CIA. Moreover, these drugs could be used also in patients who develop severe peripheral neuropathies after receiving platinum derivatives and who do not tolerate scalp cooling.

## Topical Minoxidil

Topical minoxidil 2% and 5% is widely used, but clinical studies have reported disappointing results. In particular, Rodriguez et al reported severe alopecia in 88% and 92% of the patients treated with, respectively, topical minoxidil 2% and 5% [19]. It was effective in shortening the period of baldness, as reported by Duvic et al [20], and safety and tolerability were good. Duvic et al reported scalp pruritus in 60% and scalp folliculitis in 25% of the patients, and new hair growth on the face in 8 women [20].

We would not recommend minoxidil for CIA prevention, not only because of its clinical inefficacy, but in particular because of the lack of a scientific rationale for its use.

Even if minoxidil’s mechanism of action is still not completely clear, it is well known that it induces vasodilation by opening potassium channel localized on smooth muscular cells of peripheral arteries, with consequent slowing of circulation. This leads to a greater permanence of the anticancer drug around the hair follicle. Minoxidil, in addition, is able to induce angiogenesis by upregulating vascular endothelial growth factor expression, and to activate prostaglandin endoperoxide synthase 1, which is able to stimulate hair growth [21]. This effect is exactly opposite to the scalp-cooling effect. Moreover, the final effects of minoxidil are the shortening of the telogen phase and extension of the anagen phase. Since the anagen phase is the most susceptible to drug insult, it is clear that this molecule cannot be used during chemotherapy administration.

We suggest using minoxidil after chemotherapy discontinuation in order to obtain a greater regrowth. The best moment for introducing minoxidil should be evaluated considering chemotherapeutic drug half-life and monitoring scalp with trichoscopy and trichogram [22]. Topical hydrocortisone could be associated with minoxidil topical application, acting as an anti-inflammatory agent but also helping follicular growth.

## Prostaglandin Analogue

Another proposed molecule is the topical bimatoprost 0.03%, which is a prostaglandin analogue. Its efficacy was demonstrated with a randomized trial on 130 patients affected by chemotherapy-induced or idiopathic alopecia of eyelashes, which reported a clinical improvement in 37.5% of patients vs 18.5% of controls [23]. Bimatoprost works by protecting follicles in the anagen phase and improving follicular growth in anagen I. For these reasons it should be used with the same indications of minoxidil.

## 1,25-Dihydroxyvitamin D3

Calcitriol (1,25-dihydroxyvitamin D3) has also been proposed as a CIA preventive agent. This molecule has several actions on keratinocytes, including DNA synthesis inhibition by blocking cellular cycle in G0/G1 phase, promoting cellular differentiation, and inhibiting Ki67 expression and cell growth [24,25]. Initially calcitriol appeared effective in preventing CIA induced by cytosine [26], but further studies conducted on patients in treatment with anthracyclines and cyclophosphamide showed no efficacy [27]. Moreover, contact dermatitis was reported after prolonged topical application of calcitriol.

Topical application could be replaced by systemic administration of vitamin D3 in the postchemotherapy phase in order to take advantage of its effects on the hair follicle morphogenesis, enhancing the effects of simultaneous topical application of minoxidil and/or prostaglandin analogues. Dosage should be evaluated on the basis of vitamin D basal blood levels, weight, and height, ensuring blood concentration between 40 and 60 ng/mL, which is considered the optimal range [28–30].

## Miscellaneous

Several other molecules have been proposed and studied for CIA prevention and treatment. Some of them did not give the expected results and thus were abandoned. Some others were studied only in vitro or in animal models, so further investigations are needed. These studies are summarized in Table 1 [31–40].

## Discussion

With the exception of scalp cooling, we actually have no effective drugs for CIA prevention. We emphasize that therapy should always be modulated for the patient and no fixed protocol should be used.

Trichoscopy and trichogram could help in understanding the type and grade of hair damage and what kind of treatment is the best option in a given moment [22]. Moreover, the treatment should consider the oncological therapy and course (which and how many cycles of chemotherapy the patient will face), because the intervention acts longer than instantly. We outline these concepts in Table 2.

Ideally, there should be sufficient time (from 2 to 4 weeks) before starting chemotherapy for administering a drug capable of inducing the telogen phase, in order to bring the hair follicle to a protected state during chemotherapy. If this is possible, after chemotherapy discontinuation molecules such as minoxidil and prostaglandin analogues could be used to accelerate the regrowth in anagen. In this way we would avoid the dramatic hair shedding that occurs 14 days after the first chemotherapy dose, and hair would be maintained for about 3 months when a telogen effluvium would occur, followed by a normal anagen without dystrophy. Obviously, this is a hypothesis, and more studies should be conducted.

## Conclusions

With this work we seek to clarify the mechanisms of action of drugs usually used in CIA prevention and treatment, indicating the positive and negative aspects of each (Table 3). A better understanding of the pathogenetic mechanism of CIA should lead to more successful treatment, and we hope that in the near future more efficacious, personalized, and targeted treatment options will be developed.

## Figures and Tables

**Table 1:** Molecules Proposed and Studied for CIA Prevention and Treatment

Molecule	Supposed Activity	Evidence	Model	Reference
α-Tocopherol	Preventing doxorubicin-induced alopecia	Clinical efficacy not demonstrated (systemic administration)	Human studies	Wood [31] Martin-Jimenez et al [32] Perez et al [33]
Cyclin-dependent kinase 2 (CDK-2) inhibitors	CIA prevention	CIA reduction in the 33%–50% (topical application)	Rat model	Davis et al [34]
Palbociclib (CDK4/6 inhibitor)	Prevention of alopecia induced by taxanes	Protection of transit amplifying and stem/progenitor cells through G1 arrest	Ex vivo organ culture model	Purba et al [35]
Interleukin 1	Protection from cytosine arabinoside and cytarabine-induced alopecia	CIA reduction	Rat model	Jimenez et al [36] Jimenez et al [37]
Cyclosporin	Recovery from cyclophosphamide-induced alopecia	Induction of thick and long hairs after 21 days of cyclophosphamide administration	Mice model	Shirai et al [38]
Epidermal growth factor	Protection from 1-β-D-arabinofuranosylcytosine (ARA-C)-induced alopecia	Protection from alopecia limited to the treated area	Rat model	Jimenez et al [36]
Fibroblast growth factor	Protection from 1-β-D-arabinofuranosylcytosine (ARA-C)-induced alopecia	Protection from alopecia limited to the treated area	Rat model	Jimenez & Yunis [39]
Local pharmacological inhibitors of p53	Prevention from alopecia induced by cytotoxic drugs	Just theoretical use	Real use unlikely because of possible carcinogenesis risk	Botchkarev et al [40]

In this table we summarize molecules that have been proposed and studied for chemotherapy-induced alopecia (CIA) prevention and treatment, but which are not currently used in humans and for which further investigations are needed.

**Table 2:** How Trichoscopy Could Guide Alopecia Treatment

Trichoscopic Features	Meaning	Suggested Therapeutic Conduct
Black dots and flame hairs	Acute and severe insult in anagen phase	Intensify therapies such as scalp cooling or topical vasoconstrictors with the aim of protecting hair follicles as much as possible.
Pohl-Pinkus hairs	Damage occurred, but it was not difficult to completely interrupt the mitotic activity; the insult just reduced mitotic activity (hair shaft constrictions)	The exact identification of timing could enable us to intensify the use of scalp cooling or topical vasoconstrictors when the insult is more aggressive.
Numerous yellow dots	Follicles are protected by acute damage (kenogen phase)	Use scalp cooling or topical vasoconstrictors with caution (for example, reducing the time or the number of applications). It has been hypothesized that a delayed hypoxic insult could induce neogenesis of hair follicle stem cells [15]. Indiscriminate use of scalp cooling and vasoconstrictors could be harmful if other chemotherapy cycles are expected.

**Table 3:** Studies, Positive and Negative Aspects, Probable Mechanisms of Action, and Recommendations of Available Drugs and Devices in CIA Prevention and Treatment

Available Drugs and Devices	Studies	Positive Aspects	Negative Aspects	Probable Mechanism of Action	Recommendations
Scalp cooling	Breed et al [12] Macduff et al [7] Shin et al [9] Prochilo et al [8]	High patient compliance	Headache, discomfort, nausea, and xerosis may occur Action limited to the application time	Local vasoconstriction and reduction of drug inflow to the hair follicles	Recommended for patients affected by solid tumors undergoing chemotherapeutic protocols associated with high risk of developing CIA Not recommended for patients affected by hematological tumors, cold agglutinin disease, cryoglobulinemia, and posttraumatic cold injury or receiving platinum derivatives
Topical epinephrine and norepinephrine	Soref & Fahl [15] Rathman-Josserand et al [16]	Action also during the weeks from one infusion to another Possible use also in patients receiving platinum derivatives	Application more times per day	Induction of hypoxia signal by local vasoconstriction preserves hair follicle cells and reduces the amount of drug reaching hair follicle	Recommended for patients affected by solid tumors undergoing chemotherapeutic protocols associated with high risk of developing CIA (including platinum derivatives), including patients affected by cold agglutinin disease, cryoglobulinemia, and posttraumatic cold injury Not recommended for patients affected by hematological tumors
Topical minoxidil 2% and 5%	Rodriguez et al [19] Duvic et al [20]	Good safety and tolerability Hair regrowth acceleration	No results regarding hair loss prevention	Vasodilation and angiogenesis induction (greater permanence of the anticancer drug around the hair follicle) Hair growth stimulation by activating prostaglandin endoperoxide synthase 1 Shorten telogen phase and extend anagen phase (hair follicle most susceptible to drug insult)	Not recommended for CIA prevention Recommended after chemotherapy discontinuation in order to obtain a greater regrowth (in particular in patients with previous male and female pattern hair loss)
Topical bimatoprost 0.03%	Glaser et al [23]	Good safety and tolerability Hair regrowth acceleration	No results regarding hair loss prevention	Protection of follicles in anagen phase and improving follicular growth in anagen I (hair follicle most susceptible to drug insult)	Not recommended for CIA prevention Recommended after chemotherapy discontinuation in order to obtain a greater regrowth
Topical calcitriol (1,25-dihydroxyvitamin D3)	Jimenez et al [26] Hidalgo et al [27]	None with topical application	Contact dermatitis	Action on keratinocytes	Not recommended Evaluate systemic calcitriol (1,25-dihydroxyvitamin D3) administration in postchemotherapy phase

CIA = chemotherapy-induced alopecia.
